# Editorial: Plant Competition in a Changing World

**DOI:** 10.3389/fpls.2017.00651

**Published:** 2017-04-26

**Authors:** Judy Simon, Susanne Schmidt

**Affiliations:** ^1^Ecology Group, Department of Biology, University of KonstanzKonstanz, Germany; ^2^Plant Nutrition and Ecophysiology, School of Agriculture and Food Sciences, University of QueenslandBrisbane, QLD, Australia

**Keywords:** competition, climate change, invasion, conservation, allelochemicals, global warming, facilitation, plant–plant interactions

Climate change and biological invasions place new challenges on plants, their development, fitness, and competitiveness. To develop and evaluate strategies for sustainable ecosystem management and to respond to biodiversity loss, we need mechanistic understanding of the changes that are occurring in plant communities. Underlying drivers of change are plant–plant interactions which include competition, facilitation, and avoidance of competition, and their regulation by environmental factors (Trinder et al., 2013). The studies highlighted in this ebook examine plant competition in range of communities that span from forests to meadow and crop systems across alpine, temperate, and tropical climates.

**Facilitation**, positive interactions between plant species, is a key driver of plant community dynamics and structure, but comparatively few studies have examined how facilitation is modulated in response to climate change (Brooker, 2006; Brooker et al., 2007; Lavergne et al., 2010). In their review, Anthelme et al. discuss four aspects of facilitative effects in alpine systems in response to climate change: (1) a reduction of facilitative effects in alpine plant presence in response to declining cold-temperature stress due to warming in established alpine systems, (2) an increase in facilitative effects as a response to migration to colder environments with higher elevation, (3) changing patterns of facilitation along latitudinal gradients, and (4) the potential of nurse plants to buffer changes in microhabitats. Anthelme et al. present different migration scenarios that include various types of facilitation in response to increasing temperature. Valladares et al. review the consequences of facilitation and competition in context of global change, encompassing climate change, and biological invasions, with a focus on phylogenetic relatedness, functional traits, and phenotypic plasticity. Valladares et al. summarize the direct and indirect drivers of species richness in different ecosystems, such as temperate and tropical forests, grasslands, and alpine systems. The authors argue that studying pauci-specific communities will provide the necessary understanding on species interactions in more complex systems. The reviews by Valladares et al. and Anthelme et al. conclude that although there is no doubt that climate change impacts on plant communities directly (e.g., via increasing temperatures) and indirectly (e.g., via changes in the interactions between species), further empirical knowledge is needed to advance understanding of the underlying mechanisms of plant–plant interactions in different plant communities, climate, and resource settings. For example, studies on alpine systems are biased toward certain regions, especially Europe, with other regions overlooked. The authors recommend examining systems in which single species and their intra-specific functional variability are important to expand from the current focus on species-rich systems such as tropical rainforests.

The other key driver discussed here is **competition**, especially in resource-poor habitats where plant growth and reproduction is challenging and/or further impaired with global change. A plant's ability to occupy space influences its ability to access resources such as light, water, and nutrients. Studying the relative influence of topography, environment, and spatial distance of rockcress (*Boechera stricta*) to other individuals, either intra- or inter-specific, Naithani et al. found that this species' performance in a meadow community is predominantly influenced by intra-specific competition and insect herbivory. In contrast, its spatial distribution in the meadow community is limited by dispersal and microhabitat preference. At the other end of the plant size spectrum and focusing on competition for light, van Kuij et al. present a 3D-model, validated with field data, for calculating photosynthesis rates for individual trees in forests. Such model has potential to assist forest management strategies, such as aiding the potential effect of accelerated succession to generate resilient forests/plantations.

Prior and Bowman investigated the interaction between tree growth and microhabitat across a macro-ecological gradient. They present new evidence from an extensive dataset of eucalypt tree growth collected across temperate and sub-tropical mesic Australia that in cooler habitats with sufficient water availability, light is the most limiting resource which results in increased competition, whereas in hot and dry habitats where water is the limiting factor, light is no longer driving competition. The study by Muller et al. on species interactions in urban plantings at three buildings in subtropical Australia, expands on the relationships of light and water, and demonstrates that plant productivity and arthropod diversity increase in situations with abundant availability of resources. This study provides evidence that ecological principles are transferrable from natural systems to human-made urban systems. Expanding on plant–plant competition from light and water, the study by Li et al. examines nitrogen as a main macronutrient that limits plant growth in many plant communities, and demonstrate that competition is reduced in two co-occurring tree species, beech and sycamore maple, that have a preference for organic and inorganic nitrogen forms, respectively. Another mechanism to avoid competition is allelopathy, the release of plant-growth inhibiting or toxic substances into the rhizosphere. Asaduzzaman et al. identified potentially allelopathic compounds in a laboratory bioassay investigating root and shoot tissue of different canola cultivars when growing in competition with weeds. The authors suggest that an allelopathic effect depends not only on the synthesis of certain compounds, but also on their active exudation into the rhizosphere and this seems to be dependent on intrinsic genotypic factors.

**Plant invasions** and their contribution to the competition for resources in native plant communities were reviewed by Gioria and Osborne. The authors discuss how “winning” the competition depends on factors that include resource distribution and stage of the invasion process, and that raise conceptual and methodological issues for future studies on competition in plant invasions. Considering environmental, such as competition for nutrients, water, light, and space, as well as biotic constraints, they find “windows of opportunity” during which competition is reduced. Furthermore, Gioria and Osborne show seasonal shifts between environmental or biotic constraints as key drivers of competition. Plant invasions and their role in plant–plant competition for resources has also been the focus of several original research articles here. Elgar et al. showed facilitation can provide a measure to overcome competition between native woody plants and invasive grasses in rainforest reforestation on former agricultural land. Ens et al. used leaf-scale ecophysiological and stand-scale growth traits between an invasive and a native grass and present evidence that the higher photosynthetic nitrogen use-efficiency of the invasive grass selects for improved nitrogen acquisition from soil in nitrogen-poor ecosystem. Exploring the foraging responses and performance of herbaceous invaders to nitrogen-rich patches, Keser et al. suggest that strong plasticity of root-foraging responses is adaptive and appears to contribute to invasiveness.

Overall, plant–plant competition and facilitation present a framework for understanding changes in plant communities. Such interactions are likely to become more prevalent where plants have to increasingly secure resources in response to climate change. Current knowledge together with climate predictions indicate that in some habitats competition will intensify with increased abiotic stress (e.g., water and nutrient limitations). Adding biotic stresses, such as plant invasions, will further impact on native plant communities with outcomes including declining biodiversity and ecosystem function. To date, different empirical approaches have mainly been used separately; however, using them in combination would increase resolution (Valladares et al.). Including multiple potential drivers of plant interactions in combinations in future studies, would aid in developing and evaluating strategies for sustainable ecosystem management to secure ecosystem services for modern society.

## Author contributions

JS wrote the first draft of the editorial, both JS and SS then jointly edited the final version.

### Conflict of interest statement

The authors declare that the research was conducted in the absence of any commercial or financial relationships that could be construed as a potential conflict of interest.
